# Editorial: Inflammatory Mechanisms of Hemolytic Diseases

**DOI:** 10.3389/fimmu.2021.834527

**Published:** 2022-01-12

**Authors:** Renata Sesti-Costa, João Luiz Silva-Filho, Wilma Barcellini, Caroline Le Van Kim, Nicola Conran

**Affiliations:** ^1^Hematology and Hemotherapy Center, University of Campinas (UNICAMP), Campinas, Brazil; ^2^Wellcome Centre for Molecular Parasitology, Institute of Infection, Immunity and Inflammation, University of Glasgow, Glasgow, United Kingdom; ^3^Hematology Unit, Fondazione Istituto di Ricovero e Cura a Carattere Scientifico (IRCCS) Ca’ Granda Ospedale Maggiore Policlinico Hospital, Milan, Italy; ^4^Université de Paris and Université des Antilles, INSERM, BIGR, Paris, France

**Keywords:** hemolysis, inflammation, anemia, DAMP, immune response

Hemolytic diseases have several underlying causes and result in reduced red blood cell (RBC) counts due to the destruction of these cells in the circulation. Under homeostatic conditions or mild hemolysis, free hemoglobin (Hb), heme or iron, released by RBCs in the intravascular environment, are neutralized by haptoglobin, hemopexin and transferrin, respectively. These plasmatic complexes are cellularly interiorized, degraded and the iron is recycled for use in new erythroblasts. However, during acute or chronic hemolytic diseases, intravascular scavenger molecules saturate resulting in the accumulation of free RBC components, which are potent danger-associated molecular patterns (DAMPs) sensed by endothelial and innate immune cells that trigger a cascade of oxidative and inflammatory processes. This Research Topic assembles 13 original research and 7 review articles that focus on the mechanisms inducing inflammation and how it contributes to the pathology of acute and chronic hemolytic diseases in human patients and animal models.

Recognized experts in the field contributed review articles to this issue that shed light on the heme-induced mechanisms that lead to tissue damage and disorders. Bozza and Jeney meticulously reviewed the mechanisms by which Hb-derived DAMPS, such as free heme, metHb and ferrylHb, act as proinflammatory triggers. They elucidate the cascade of Hb and heme oxidation, which leads to reactive and damaging molecules, and clarify the knowledge gathered so far about the ligands and the pathways that directly stimulate the activation of endothelial and innate immune cells, promoting endothelial barrier permeability, leukocytes recruitment, secretion of proinflammatory cytokines, the generation of reactive oxygen species (ROS), neutrophil extracellular traps (NET) formation, cell death and trained immunity. Jeney et al. reported that oxidized Hb forms, free heme and a product of heme catabolism, bilirubin, were found in the cerebrospinal fluid from infants with intraventricular hemorrhage (IVH), which correlated with VCAM-1, ICAM-1 and IL-8 levels. The direct effect of Hb products was shown by the treatment of a human brain endothelial cell line with heme, which induced significant production of ROS and decreased cell viability. Furthermore, treatment with either heme or ferrylHb up-regulated VCAM-1, ICAM-1 and IL-8 expression, which was not achieved by native Hb or even metHb, revealing specific functions of the different products of oxidized Hb in endothelial cells.

Pádua and Souza focused their review on the role of heme in pulmonary malaria, a disease characterized by the presence of free heme and heme oxidation due to hemolysis caused by RBC-infected *Plasmodium* sp. The system involved in the clearance of heme has been shown to play a protective role in malaria outcomes, since the level of hemeoxygenase-1 (HO-1), the enzyme responsible for heme detoxification, is positively correlated with protection from multi-organ dysfunction and survival. A peculiar feature of plasmodium infection is the hemozoin, a bio-crystallized structure produced by the parasites after digestion of Hb from infected-RBCs, thus another form of heme derivative that is not present in other hemolytic disorders. Some data show that hemozoin is phagocyted together with the RBCs content mainly by macrophages and monocytes, which become activated to produce proinflammatory cytokines and chemokines, leading to pneumocyte apoptosis and loss of alveolar integrity.

Free heme and iron are extremely oxidative, generating ROS in the vascular milieu by different mechanisms. This oxidative stress has a direct cytotoxic effect, causing the oxidation of proteins, lipids, and DNA, which culminates in tissue damage, as well as an indirect effect by generating and releasing new DAMPs. Thus, ROS seem to be a key player in a great feedforward loop that is activated by RBC products and, in turn, is able to generate even more reactive mediators, amplifying the inflammatory response. This can be exemplified by sickle cell disease (SCD), as widely reviewed by Connes’s and Kato’s groups. As precisely covered by Nader et al., SCD is a disease characterized by a vicious circle between abnormality in RBCs and inflammation. A genetic single mutation in the β-globin gene leads to an anomalous hemoglobin (HbS), which polymerizes under deoxygenation, changing the deformability and fragility of the RBCs and, consequently, causing intravascular hemolysis. In sequence, cell free hemoglobin and heme induce ROS production and deplete nitric oxide (NO) from the microenvironment. The pro-oxidative milieu created is responsible for oxidation of several compounds, and may further increase RBC death and inflammation, amplifying the damage. Indeed, Connes’s group (Nader et al.) demonstrated the effects of ROS and NO on RBC deformability and eryptosis. ROS was also involved in sickle RBC-derived microparticle (MP) release. The levels of circulating RBC-MPs correlated with arterial stiffness in SCD patients and were able to activate a human endothelial cell line, inducing secretion of proinflammatory cytokines through a TLR4-dependent mechanism. In line with that, Santana et al. showed that hydroxyurea (HU), a FDA approved drug for SCD, induces antioxidant gene expression, such as superoxide dismutase-1 and glutathione disulfide-reductase. Moreover, HU was able to reduce ROS production induced by hemin treatment, suggesting that HU acts by an additional and previously unappreciated role in SCD disease by inducing antioxidant proteins and, hence scavenging free radicals.

In their review article, Gbotosho et al. decipher each of the heme-induced pathways that lead to the complications of SCD, where excessive ROS are generated by RBCs due to the unstable nature of HbS and to a greater percentage of NADPH oxidase and mitochondria retention in mature RBCs. The RBCs-derived microparticles generated in SCD are also able to deliver heme to endothelial cells, mediating additional oxidative stress. Besides the role of heme as pro-oxidative mediator and a DAMP, the authors emphasize its ability to induce PlGF and IL-6 and discuss several pathways by which these mediators can contribute to the multiple pathophysiology of SCD. The direct link between free heme in the bloodstream with IL-6 production and with tissue damage is shown in a mice model by another article from the group Gbotosho et al. By injection of heme in the mice, they demonstrated, *in vivo*, the expression of IL-6 and markers of cardiac stress and hypertrophy in Townes mice, a model of SCD, through a pathway independent of Nrf2, the canonical transcription factor activated by HO-1.

Belcher and Vercellotti’s groups investigated different aspects of TLR4 signaling induced by heme. Although TLR4 is a well described heme ligand and it is widely expressed, it is not completely clear which cells are the TLR4-expressed effectors of the heme-induced proinflammatory disorders. By generating a bone marrow chimera deficient in TLR4 in a murine model of SCD, Beckman et al. elegantly demonstrated that endothelial, but not hematopoietic, TLR4 expression is necessary for the microvascular stasis presented in the disease. In order to understand how TLR4-dependent heme stimulation occurs, Belcher et al. performed a detailed investigation on this matter. Through pull-down and reporter assays they demonstrated that, similarly to LPS, heme binds to the adaptor molecule myeloid differentiation factor-2 (MD-2) to initiate NF-kB signaling, a process that is dependent on TLR4 and CD14. Interestingly, by generating MD-2 mutants, they were able to identify possible binding sites for heme in the MD-2 molecule, which they found to be different to the binding sites for LPS. Although MD-2 is often co-expressed with TLR4 on the cell surface, Zhang et al. showed high levels of the soluble form of MD-2 (sMD-2) in human and murine sickle plasma, which was shown to induce IL-8 secretion by human endothelial cells, suggesting that heme bound to sMD-2 is also able to promote endothelial cell activation in SCD.

Santaterra et al. showed that besides the induction of inflammatory cytokines, heme present in the serum of SCD patients promotes endothelial barrier disruption *in vitro* in a manner dependent on hemopexin levels in serum, indicating that when the free heme clearance system is overwhelmed, heme is able to cause injury. In line with this observation, Poillerat et al. demonstrated the pharmacokinetic properties of hemopexin in mice in order to guide future therapy for hemolytic diseases by scavenging free heme from the bloodstream.

Neutrophils and monocytes are recognized as major producers of inflammatory cytokines in the vascular microenvironment during hemolysis. However, other cells may play a role in this process. As evidence of this, HO-1was shown to be involved in inflammatory dendritic cell (DC) differentiation from circulating monocytes by Sesti-Costa et al., suggesting a role for heme or its derivatives in the generation of these immune cells. Accordingly, SCD patients had higher circulating inflammatory DCs, and monocyte-derived DCs from patients were able to produce MCP-1, IL-6 and IL-8 in culture and induce Th17 and Tc17 profile skewing, indicating that hemolysis may also alter the response of a previously unappreciated immune cell subset.

The inflammatory cytokine and chemokine landscape of SCD patients in different statuses was carefully analyzed by Silva-Junior et al., who observed that patients have a proinflammatory profile even in steady state. Interestingly, some of the secreted mediators are decreased upon vaso-oclusive crisis (VOC), which is mainly marked by cytokines involved with lymphocyte proliferation, Th2 responses, and high levels of G-CSF, an important growth factor for neutrophil development. PDGF-BB and IL-1ra were pointed out as important hallmarks of clinical recovery post-VOC, as they are strikingly high during crisis and were the ones that significantly declined in the convalescent stage, representing potential markers for prognosis. A novel potential approach for managing SCD is also brought by Kiven et al. with a new way to evaluate pain status in SCD. Using an automated gait measurement method, the authors accessed gait patterns in a mouse model of SCD and found alterations in stance instability and spatial and temporal gait parameters. Interestingly, changes in gait correlated with mechanical and deep tissue hyperalgesia. In addition, purkinje cells, which are associated with movement and neural function, were shown to undergo apoptosis in the cerebella of SCD mice, suggesting that neuronal damage and chronic pain may incite compensatory gait alterations in SCD.

Two complete reviews in this Research Topic bring novel insights on the landscape of autoimmune hemolytic anemia (AIHA). Barcellini and Fattizzo underscored the heterogeneity of the disease, as the pathology depends on the isotype and complement-induced ability of the autoantibodies specific to the RBCs developed. The outcome is also reflected by the effectiveness of the compensatory erythropoiesis and whether the cause is either primary or secondary. The authors critically discuss the currently available therapies, the ones in development and the challenges encountered to obtain accurate treatment and diagnosis. In line with the underappreciated heterogeneity of the disease, the prevalence of a PNH clone in AIHA patients was found to be higher than expected (37.4%) by Fattizzo et al. In addition, the hemolytic pattern and cytokine profile differed between PNH positive versus negative in AIHA patients, suggesting that they may be in need of different therapy indications and that testing for PNH may be beneficial especially in cases unresponsive to current AIHA treatment.

The differences in the pathogenesis of AIHA mediated by cold agglutinins is another instance of the heterogeneity of outcomes in the disease, as covered by Berentsen. In this case, cold-reactive antibodies that are able to agglutinate RBCs arise from either a clonal lymphoproliferative source, such as in cold agglutinin disease (CAD), or a secondary clinical disorder, as seen in secondary cold agglutinin syndrome (CAS). The author extensively reviews the immune pathogenesis of both diseases, covering the origin and role of the agglutinins, the activation of complement and induction of inflammation, which considerably impact the chosen treatment. Finally, Zaninoni et al. discuss the poorly studied role of the immune system in congenital hemolytic anemia (CHA), especially the levels of naturally occurring antibodies that can play different roles in health and disorders, and are involved both positively and negatively in autoimmune disease. The authors highlighted the evidence so far that suggests that these antibodies may have a pathogenic role in CHA by inducing removal of RBCs in the spleen, and also the implications of splenectomy for the immune response in CHA patients.

We hope the readers enjoy this Research Topic issue that contains a relevant update on the consequences of hemolytic anemia from different causes, and we thank all authors that provided their valuable contributions to this collection.

## Author Contributions

All authors contributed to the article and approved the submitted version.

## Funding

RS-C and NC are funded by FAPESP, grant numbers 2019/09704-7 and 2021/05191-5 granted to RS-C, and 2019/18886-1 granted to NC.

## Conflict of Interest

The authors declare that the research was conducted in the absence of any commercial or financial relationships that could be construed as a potential conflict of interest.

## Publisher’s Note

All claims expressed in this article are solely those of the authors and do not necessarily represent those of their affiliated organizations, or those of the publisher, the editors and the reviewers. Any product that may be evaluated in this article, or claim that may be made by its manufacturer, is not guaranteed or endorsed by the publisher.

